# Leaf Conditioning and Shredder Activity Shape Microbial Dynamics on Fine Particulate Organic Matter Produced During Decomposition of Different Leaf Litter in Streams

**DOI:** 10.1007/s00248-025-02515-2

**Published:** 2025-03-22

**Authors:** Pratiksha Acharya, Mourine J. Yegon, Leonie Haferkemper, Benjamin Misteli, Christian Griebler, Simon Vitecek, Katrin Attermeyer

**Affiliations:** 1 Wassercluster Lunz – Biological Station, Dr. Carl Kupelwieser-Prom. 5, 3293 Lunz Am See, Austria; 2 Department of Functional and Evolutionary Ecology, Unit Limnology, University of Vienna, Djerassiplatz 1, 1030 Vienna, Austria; 3 Institute for Hydrobiology and Water Management (IHG), University of Natural Resources and Life Sciences, Gregor-Mendel-Straße 33/DG, 1180 Vienna, Austria; 4 Department of Aquatic Ecology, Eawag, Swiss Federal Institute of Aquatic Science and Technology, Überlandstrasse 133, 8600 Dübendorf, Switzerland; 5 Department of Ecology, University of Innsbruck, Technikerstraße 25, 6020 Innsbruck, Austria

**Keywords:** Leaves, Faecal pellets, Microbial assemblage, FPOM composition, Microbial activity

## Abstract

Leaf litter decomposition (LLD) is a key ecosystem function where invertebrate shredders produce large amounts of fine particulate organic matter (FPOM) that serves as a substrate for microbial assemblages. Here, we explore the shredder-produced FPOM composition and activity of FPOM-associated microbial communities in response to different leaf species and their conditioning. In a laboratory experiment, we fed leaves of different elemental compositions (alder, beech and maple), conditioned under oxic or anoxic conditions, to caddisfly larvae (S*ericostoma* sp.). We hypothesized differences in FPOM elemental and fatty acid composition and FPOM-associated microbial activity among the leaf species, conditioning, and two types of shredder-produced FPOM, i.e. shredded leaves and faecal pellets. Our results suggest that leaf conditioning and shredder activity play pivotal roles in shaping FPOM composition and FPOM-associated microbial activity. We observed lower C/N ratios with high-C/N litter (beech and maple leaves) after conditioning and no change in the elemental composition of the faecal pellets compared to the leaves. However, we observed differences in microbial fatty acid proportions and composition on leaves and faecal pellets with significantly higher fractions of bacterial fatty acids on faecal pellets than on leaves. We also noted a significant impact of leaf conditioning on the microbial activity of shredded leaves and faecal pellets, with a higher microbial growth efficiency observed on faecal pellets compared to ingested leaves. These findings highlight the crucial influence of leaf species and conditioning on the activity of shredder-produced FPOM, emphasizing the complex interplay between leaf properties and fate and microbial processes in streams.

**Supplementary Information:**

The online version contains supplementary material available at 10.1007/s00248-025-02515-2.

## Introduction

Substantial amounts of organic matter enter headwater streams from the surrounding riparian ecosystems—primarily in the form of leaf litter [1] that is fragmented into smaller particles [2]. Leaf litter enters the streams as coarse particulate organic matter (CPOM > 1 mm) with a wide range of nutritional quality, including differences in carbon-to-nitrogen (C/N) ratios and fatty acid composition. Some types of CPOM can provide readily available nutrients, labile organic molecules and low carbon-to-nitrogen ratios (hereafter, C/N ratios). However, other CPOM contains recalcitrant compounds and is hardly degradable with high C/N ratios. In all cases, fresh CPOM entering streams is first colonized by aquatic microbial communities. The rate of microbial colonization and community composition depends on the above-mentioned leaf properties and the environmental conditions. This process is known as “conditioning” [3, 4] which reduces the C/N ratio and thereby enhances the nutritional quality of the leaves [5, 6]. Furthermore, microorganisms colonizing the leaves can synthesize specific fatty acids (FAs), including linoleic acid (LIN, 18:2n-6), α-linolenic acid (ALA, 18:3n-3), palmitoleic acid (16:1n-7c), vaccenic acid (18:1n-7c) and oleic acid (18:1n-9) [7]. These FAs are typically biomarkers for aquatic hyphomycetes (e.g. LIN) and heterotrophic bacteria, which further help aquatic consumers meet their nutritional requirements. In general, microbial growth on leaves can facilitate the subsequent CPOM consumption by invertebrate shredders [3, 8]. As shredders consume CPOM, they produce fine particulate organic matter (FPOM < 1 mm) via two pathways: as small leaf litter fragments during the shredding of the leaves, also known as sloppy feeding [8, 9] and as faecal pellets during defecation [9, 10]. These two types of FPOM significantly contribute to stream ecosystem functioning, with faecal pellets accounting for up to two-thirds of FPOM in headwater streams [10], forming an essential component of the organic matter budget [11]. However, most of the existing studies on leaf litter decomposition (LLD) have focused on CPOM decomposition by shredders and microorganisms, whereas shredder-produced FPOM types and their associated microbial communities have been largely neglected.

Previous studies addressing both terrestrial and aquatic ecosystems revealed environmental conditions and CPOM quality as the main drivers of LLD [12, 13]. Environmental conditions can affect the LLD both directly by altering temperature and nutrients and indirectly by altering the CPOM quality and/or the decomposer community composition [14]. In streams, dissolved oxygen concentrations fluctuate spatially, leading to oxic conditions in flowing or turbulent sections and anoxic conditions in hyporheic sediments or slow-flowing sections or stagnant pools where leaves accumulate during autumnal leaf fall on stream beds [15, 16]. This in turn influences CPOM processing [17]. Oxic waters typically support higher microbial biomass and activities, including eukaryotes that potentially boost CPOM decomposition [18]. In contrast, anaerobic metabolic processes, i.e. fermentation and anaerobic respiration in anoxic zones, are mostly driven by bacteria-dominated microbial communities and characterized by low microbial activity [18, 19]. Thus, indirect effects of stretch-scale differences in oxygen availability, in combination with primary qualitative indicators such as CPOM C/N ratios, may influence LLD by shredders, shredder-produced FPOM types and their composition. Nevertheless, the effects of direct and indirect qualitative differences of CPOM on the shredder-produced FPOM-associated microbial activity have not been well described yet. These effects are not included in the current conceptual understanding of LLD in streams.

Shredder-produced FPOM has been described and investigated as an important resource for filter feeders [20, 21]. However, the shredder-produced FPOM also serves as a food source as well as a physical habitat [22, 23] for microorganisms. FPOM-associated microbial communities play a key role as FPOM decomposers [24]. Additionally, their colonization ultimately alters the nutritional value of FPOM by decreasing the C/N ratio [5, 25] and thus contributes to energy transfer and organic matter turnover in the stream’s food web [26]. Yet, if and how shredder activity influences FPOM composition and activity of FPOM-associated microbial communities remains to be investigated.

In this study, we aimed to test whether CPOM C/N ratios are mirrored in shredder-produced FPOM and if and how these compositional differences influence the activity of FPOM-associated microbial communities. Therefore, we conducted a lab experiment to investigate how the CPOM composition, manipulated through leaves of different deciduous tree species, and oxygen availability, i.e. either oxic or anoxic conditioning of these leaves, affect shredder-produced FPOM properties and FPOM-associated microbial communities. We specifically focused on the protein production and respiration of FPOM-associated microorganisms as a biotic response of FPOM composition and considered the two types of shredder-produced FPOM—FPOM from mechanically shredded leaves, mimicking the feeding behaviour, and faecal pellets. We hypothesized that (i) C/N ratios differ between oxic- and anoxic-conditioned leaves and the shredded leaves versus the faecal pellets; (ii) fatty acid composition differs between the conditioned leaves and the two types of shredder-produced FPOM, i.e. shredded leaves and faecal pellets. These compositional differences should be reflected in the microbial activities and we additionally hypothesized that (iii) microbial activities differ among the leaf species, leaf conditioning and the two types of shredder-produced FPOM.

## Materials and Methods

We carried out a 25-day feeding experiment in indoor microcosms (12 °C, 11:13 light:dark period) with a single shredder species (Trichoptera: *Sericostoma* sp.) fed with anoxic- and oxic-conditioned leaves of different riparian tree species. We collected shredder-produced FPOM and analyzed its composition as well as the activity of FPOM-associated microbial communities.

### Experiment Preparation

We collected naturally abscised leaves around Lunz am See, Austria (47°51′ N, 15°04′ E) during the period of maximal leaf fall in October 2021. We selected three tree species commonly found in Central Europe covering a range of CPOM quality: alder (*Alnus glutinosa* (L.) Gaertn.), beech (*Fagus sylvatica* L.) and maple (*Acer platanoides* L.), where alder serves as our high-quality CPOM and maple and beech as lower quality CPOM based on C/N ratios (supplementary; Table S1). We dried the collected leaves at room temperature and stored them in dry and dark conditions. For the experiment, we cut leaf discs of 18 mm in diameter from all three leaf species using a cork borer.

We packed 0.802 ± 0.003 (mean ± SD) g leaf discs of each leaf species in mesh bags (1 mm mesh size) for either anoxic or oxic conditioning. For anoxic conditioning, we immersed the mesh bags in clay slurry (unfired pottery clay; Glorex AG, Füllinsdorf, Switzerland) mixed 1:1 with 500 µm hand-sieved stream water from Oberer Seebach (OSB), a relatively pristine second-order forested stream located in the pre-alpine region around Lunz am See, for 4 weeks. The mixture was gently shaken every 3 days. Before the start of the experiment, we gently washed the conditioned leaves with filtered stream water to remove the excess sediments. For oxic conditioning, we submerged the mesh bags in freshly collected 500 µm-filtered stream water from OSB, which was continuously aerated. We kept both conditioning set-ups in the dark at 12 °C and monitored the dissolved oxygen concentration (WTW Oxi 315i; Xylem Analytics, Weilheim, Germany) in both conditioning treatments to confirm oxic (8.4 ± 1.0 mg L^−1^) and anoxic (below 1.3 ± 0.5 mg L^−1^) conditions, respectively. Despite measuring low concentrations of oxygen in the clay slurry, we refer to this treatment as “anoxic” as slight dissolved oxygen introduction likely occurred during the electrode movement, and additional analyses of the bacterial community on the anoxic-conditioned leaves show that the biofilm was dominated by obligate anaerobic bacteria (data not shown). We took subsamples of all leaf species immediately after termination of conditioning for analyses of CPOM quality as well as a baseline for microbial response assessments.

We collected *Sericostoma* sp. larvae of similar size (head capsule width 2.0 ± 0.1 mm) from Unterer Seebach (USB; 47°51′29″ N 15°2′4.74″ E) and acclimated them in separate food-grade plastic cups (polylactic acid (PLA), volume 4 cl) at 12 °C in filtered stream water (0.7 µm, 450 °C, 4 h precombusted Whatmann GF/F filters). Larvae were starved for 24 h before the start of the feeding experiment.

### Microcosm Setup

Each microcosm consisted of a food-grade white plastic bucket (1100 mL volume and 122 mm bottom diameter), a steel mesh (pore size 1 mm) installed at a height of 4.5–5 cm from the bottom and 750 mL stream water. We added ten shredders to each microcosm and fed them with different conditioned leaves, so that we obtained a total of 30 microcosms (3 leaf species × 2 conditionings × 5 replicates = 30). We collected the faecal pellets on the 5th, 15th and 25th day (5d, 15d and 25d) by separating each larva from each microcosm for 24 h in a plastic cup with filtered stream water before returning them to the microcosms. However, we pooled pellets from two larvae from each microcosm to have enough material for our measurements, providing us with five samples per microcosm and sampling. We analyzed bacterial protein production (BPP) and microbial respiration (MR) immediately, while samples for particulate organic carbon (POC), particulate nitrogen (PN) content and fatty acid (FA) composition were stored at − 80 °C until further processing. We also collected the leftover leaf discs at the end of the experiment for POC, PN and FA analyses and stored them at − 80 °C until further processing (see details in supplementary).

### FPOM Composition

We measured the C and N concentrations in freeze-dried (Virtis™ Genesis Freeze Dryer, for minimum 24 h) leaf samples and faecal pellets on a Flash HT Plus CHNS/O elemental analyzer (Thermo Fisher Scientific, Bremen, Germany). Additionally, we analyzed the FA composition after lipid extraction and derivatization to fatty acid methyl esters (FAMEs) that were quantified as FAMEs using gas chromatography (GC) following the procedures described in Guo et.al [27]. We identified 47 single FAs using reference standards and calibration curves and more details on both procedures can be found in the supplementary material. For both analyses, faecal pellets per two larvae per microcosm were insufficient, so we pooled two larvae’s faecal pellets from five microcosms for each treatment (faecal pellets of 2 larvae × 5 microcosms per treatment = faecal pellets from 10 larvae), resulting in a single sample per treatment for both analyses.

### FPOM-Associated Microbial Activities

We incubated finely cut anoxic or oxic conditioned leaf discs from different leaf species, here referred to as "shredded leaves," and pooled faecal pellets from two larvae per microcosm for MR and BPP measurements. We quantified respiration in our samples via oxygen consumption over time with a needle-type O_2_ microsensor (Microx 4, Presens GmbH, Regensburg, Germany). We converted the amount of consumed O_2_ to µg C L^−1^ d^−1^ by using a conversion factor of 1 [28]. Here, we express MR for shredded leaves and faecal pellets as µg C h^−1^ gDW^−1^ and ng C h^−1^ 2 animals^−1^, respectively. In addition, we determined BPP via the incorporation of L-Leucine [4,5-^3^H] into the protein fraction using the protocol of [29] and [30]. We reported BPP as µg h^−1^ gDW^−1^ for shredded leaves and ng h^−1^ 2 animals ^−1^ for faecal pellet samples (see details in supplementary).

### Data Analysis

To test our first hypothesis on differences in C/N ratios between anoxic- versus oxic-conditioned leaves and between the two types of shredder-produced FPOM, we used non-parametric Wilcoxon signed-rank tests (function *wilcoxon.test*; *stats* package). In the first test, we compared C/N ratios from anoxic versus oxic conditioning paired by each leaf species (three pairs), while in the second test, we compared C/N ratios of leaves versus faecal pellets paired by the treatments and included data from the first and last faecal pellet sampling (12 pairs). Due to the small number of comparisons, we ran non-parametric tests here.

To test our second hypothesis and compare the FPOM FA composition between different groups, relative proportions (%FAME) of the most abundant single FAs (> 1% of total FAME) in leaves and faecal pellets as well as the FA categories omega-3 polyunsaturated fatty acids (n-3 PUFA) and sum of all bacterial fatty acids (BFA) were used. We first conducted a Principal Component Analysis (PCA) (function *prcomp*; *stats* package) to visualize compositional differences in FA profiles between the leaves and faecal pellets based on the proportional FAME data (*ggplot2* package) [31] in R [32] after the variables had been scaled. Ellipses represented 70% intervals around centroids of collected FPOM. We also applied non-parametric multivariate analysis of variance (PERMANOVA) on the most abundant single FAs to assess the effects of conditioning, FPOM types and their interaction using “Mahalanobis” dissimilarities as a distance measure (function *adonis2*; *vegan* package). The assumption of homogeneous within-group dispersion was tested (function *betadisper; vegan* package) and was fulfilled for all groups.

For our third hypothesis regarding the differences in microbial activities between FPOM type, leaf species and conditioning, we conducted several tests. For the MR and BPP of the shredded leaves, we ran a linear model using leaf species and conditioning as fixed factors. For the MR of the faecal pellets, we tested the differences between leaf species, conditioning, time and their interactions as fixed factors and microcosm ID as a random factor in a linear mixed model (function *lmer*; *lme4* package) [33], aiming to account for potential variation among microcosms. The dataset for the BPP of faecal pellets is too small and does not permit the use of linear mixed models; therefore, we tested for effects of leaf species and conditioning in a simple linear model (see supplementary methods description for more information).

Finally, we assessed differences in microbial growth efficiency (MGE) by running a linear model (function *lm*; *stats* package) using FPOM type, leaf species and conditioning as fixed factors. We calculated MGE (in %) for both shredded leaves and faecal pellets as [34]:

1 $$\mathrm{MGE}=\frac{\mathrm{BCP}}{\mathrm{MR}+\mathrm{BCP}} \times 100$$

where BCP = bacterial carbon production and MR = microbial respiration. Since the bacterial bulk C to bulk protein ratio is 0.86 on average, we calculated BCP from BPP by multiplying it by 0.86 [29]. We calculated the MGE to allow comparisons of MR and BPP among FPOM types, as measurements on faecal pellets could not be based on the dry weight of organic matter. Therefore, we now have the standardized units.

Afterwards, we ran an analysis of variance (ANOVA) to evaluate each model fit and report the significant factors. Further, we calculated the effect size (function *effectsize*; *effectsize* package) [35] after running each model fit to identify the strongest influencing fixed factor. We checked the goodness of fit of all the MR, BPP and MGE models (normality of residuals, homogeneity of variance and collinearity) using graphical procedures and Shapiro–Wilk tests. Data transformation (i.e. log, square root) was performed wherever necessary to meet the assumptions. We calculated the estimated marginal means and the upper and lower limits of the 95% confidence interval (function *emmeans; emmeans* package) [36] and reported adjusted *p*-values after using a multivariate *t*-distribution approach for pairwise comparisons among the groups (function *pairs*; *emmeans* package).

All parameters are reported as means ± standard deviation throughout the manuscript; otherwise, they would be mentioned individually. For all the data analyses and visualizations, we used R 4.2.3 [32].

## Results

### Elemental Compositional Change in Shredded Leaves and Faecal Pellets

**Fig. 1:**
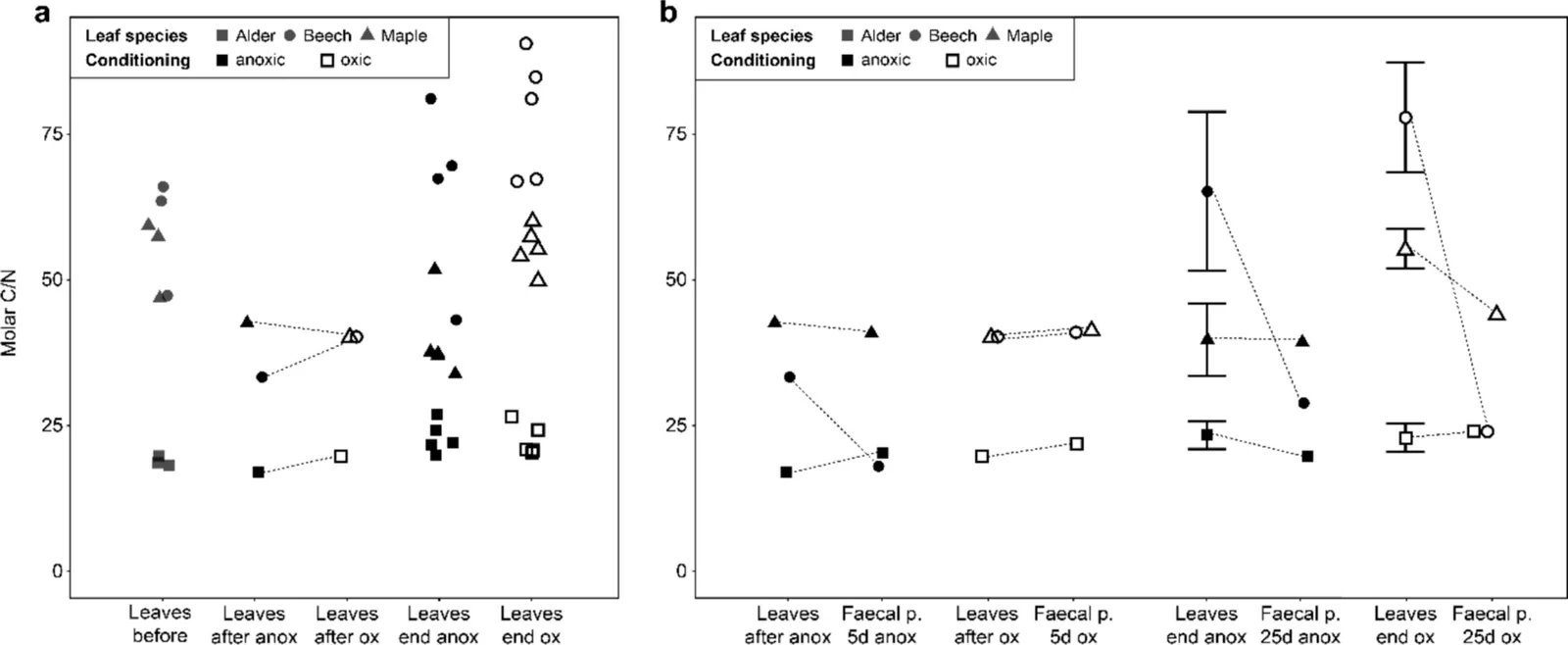
Molar C/N ratios of leaves for all sampling times (**a**) and of leaves and faecal pellets from first and last sampling (**b**). In **a**, data are shown for the originally collected leaves (before), the leaves directly after anoxic or oxic conditioning (after anox or after ox) with paired comparisons used for testing changes between anoxic and oxic conditioning (indicated by dashed lines connecting datapoints) and the leaves at the end of the 25-day feeding experiment (end) with individual values for information. In **b**, data are shown for the start leaves and 5d faecal pellets for anoxic and oxic conditioning, as well as the 25d leaves (= end) and faecal pellets for anoxic and oxic conditioning. The dashed lines connecting datapoints indicate paired comparisons between time points. The mean of the five replicates of leaves (displayed as mean with standard error) was used for paired comparisons with faecal pellets at the same time point. The oxic or anoxic conditioned samples are indicated by either black (anoxic) or white (oxic) and the three leaf species are shown by three different symbols

We found no differences between the C/N ratios of oxic- or anoxic-conditioned leaves (Fig. 1a; Wilcoxon signed-rank test; *V* = 1; *p*-value = 0.5; df = 2), in contrast to our hypothesis. However, both oxic and anoxic leaf conditioning decreased the molar C/N ratio of the high-C/N CPOM (58.92 to 36.78 and 54.60 to 41.36 for beech and maple, respectively) while the alder C/N ratio remained at a similar level before and after conditioning (19.12 to 18.34) (Fig. 1). At the end of the feeding experiment, the C/N ratio of shredded oxic and anoxic-conditioned beech leaves increased approximately twofold, exceeding the C/N ratio from before conditioning. In contrast to our hypothesis, we also detected mean C/N ratios in the faecal pellets that were similar to those of the conditioned leaves for alder and maple treatments, and only anoxic-conditioned beech decreased by 7.9 (Fig. 1b; Wilcoxon signed-rank test; *V* = 23, *p*-value = 0.23, df = 11).

### Fatty Acid Compositional Changes in Shredded Leaves and Faecal Pellets

**Fig. 2:**
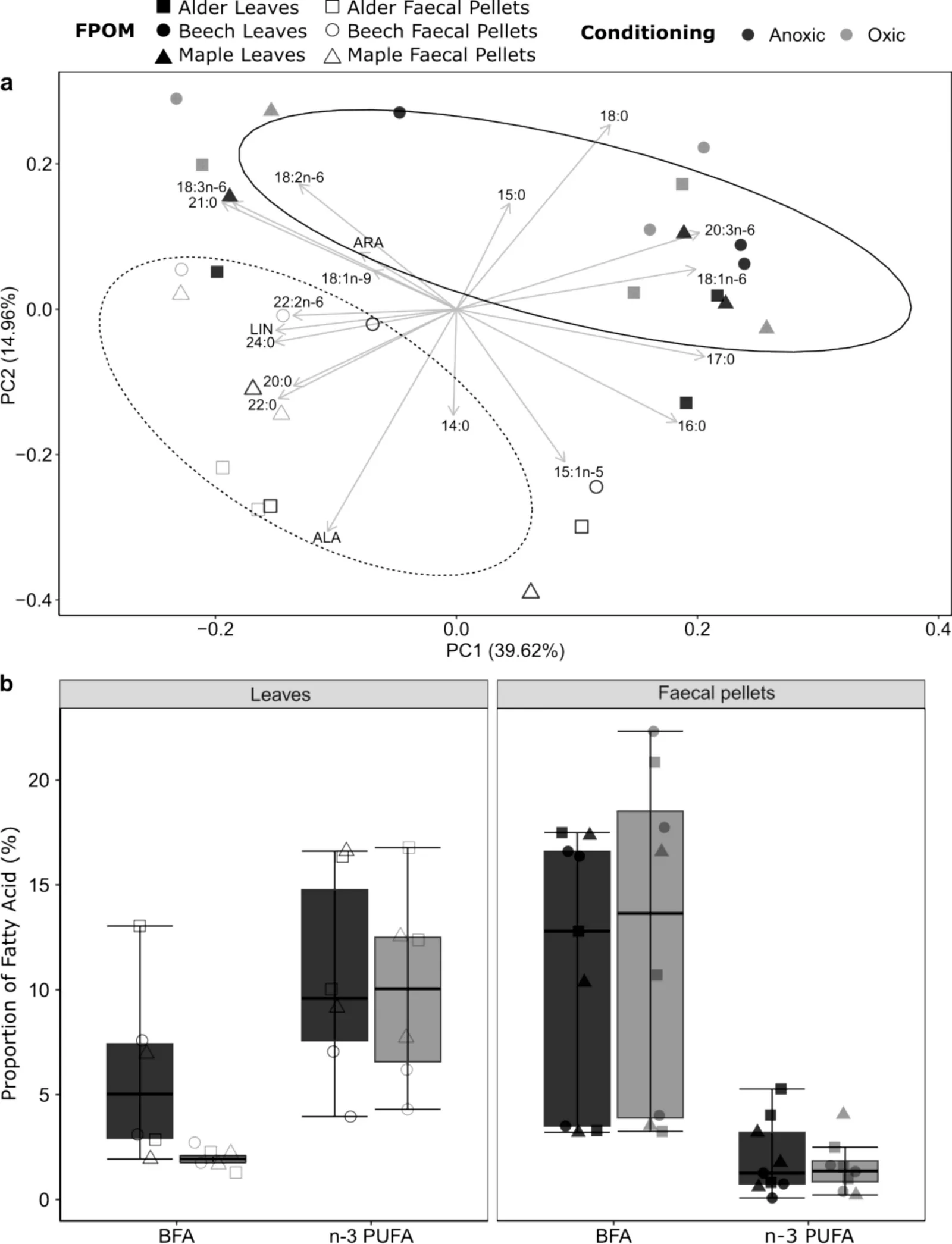
Changes in fatty acid (FA) composition of shredded leaves and faecal pellets during leaf breakdown with a bivariate plot based on a principal component analysis of all collected FPOM and their fatty acid compositions (**a**) and boxplots of relative proportions of sums of bacterial fatty acids (BFA) and omega-3 polyunsaturated fatty acids (n-3 PUFA) (**b**). All plots contain the faecal pellets data from all three timepoints and the leaves data from start and end of the feeding experiment. The two colours distinguish the samples from oxic or anoxic conditioning. The three different empty symbols indicate three leaf species, and the three different filled symbols indicate faecal pellets obtained from shredders after feeding those three leaf species. In **a**, dotted and solid line of ellipses (70% confidence interval) encircle the shredded leaves and faecal pellets collected during the experiment, respectively

The PCA of the relative proportions (% of total FAME) of the 20 most abundant individual FAs showed a clear separation between shredded leaves and faecal pellets (Fig. 2a). The PC1 accounted for 39.62% of the variation and was strongly correlated with the FAs, 17:0 (eigen values = 0.32), 20:3n-6 (0.32), 18:1n-6 (0.31), 18:3n-6 (− 0.31) and 21:0 (− 0.29), palmitic acid (16:0; 0.29), 24:0 (− 0.24), linoleic acid (LIN; 18:2n-6; − 0.24), 22:0 (− 0.23) and 22:1n-6 (− 0.21). The PC2 accounted for 14.96% of the variation and was strongly correlated with the FAs, alpha-linolenic acid (ALA; 18:3n-3; − 0.49), stearic acid (18:0; 0.40), 15:1n-5 (− 0.33), 18:3n-6 (− 0.27), 16:0 (− 0.25) and 21:0 (0.23). The PERMANOVA analysis showed that the FA composition of shredded leaves was significantly different from those of faecal pellets (Table 1). We found no difference in the FA composition of shredder-produced FPOM between oxic and anoxic conditioning (Table 1). The proportion of n-3 PUFA was six times higher on shredded leaves than on faecal pellets (10.3 ± 4.7 versus 1.7 ± 1.4%; type III ANOVA; *F*_(1,26)_ = 48.08; *p* < 0.001), while the proportion of BFA was five times higher on faecal pellets than on shredded leaves (11.8 ± 6.8 versus 2.2 ± 0.6%; type III ANOVA; *F*_(1, 26)_ = 12.19; *p* < 0.05) (Fig. 2b).

**Table 1:** Effects of FPOM type, conditioning and their interaction on FA composition as assessed by PERMANOVA. Abbreviations: SS, sum of squares; *R*^2^, coefficient of determination; df, degrees of freedom

Factors	*df*	SS	*R*^2^	*F*-value	*p*-value
FPOM types	1	27.272	0.057	1.645	**0.001**
Conditioning	1	15.651	0.033	0.967	0.160
FPOM type: Conditioning	1	19.701	0.041	1.191	0.198
Residual	25	413.375	0.868		

Statistically significant *p*-values (< 0.05) are shown in bold

### Microbial Activities of Shredded Leaves and Faecal Pellets

**Fig. 3:**
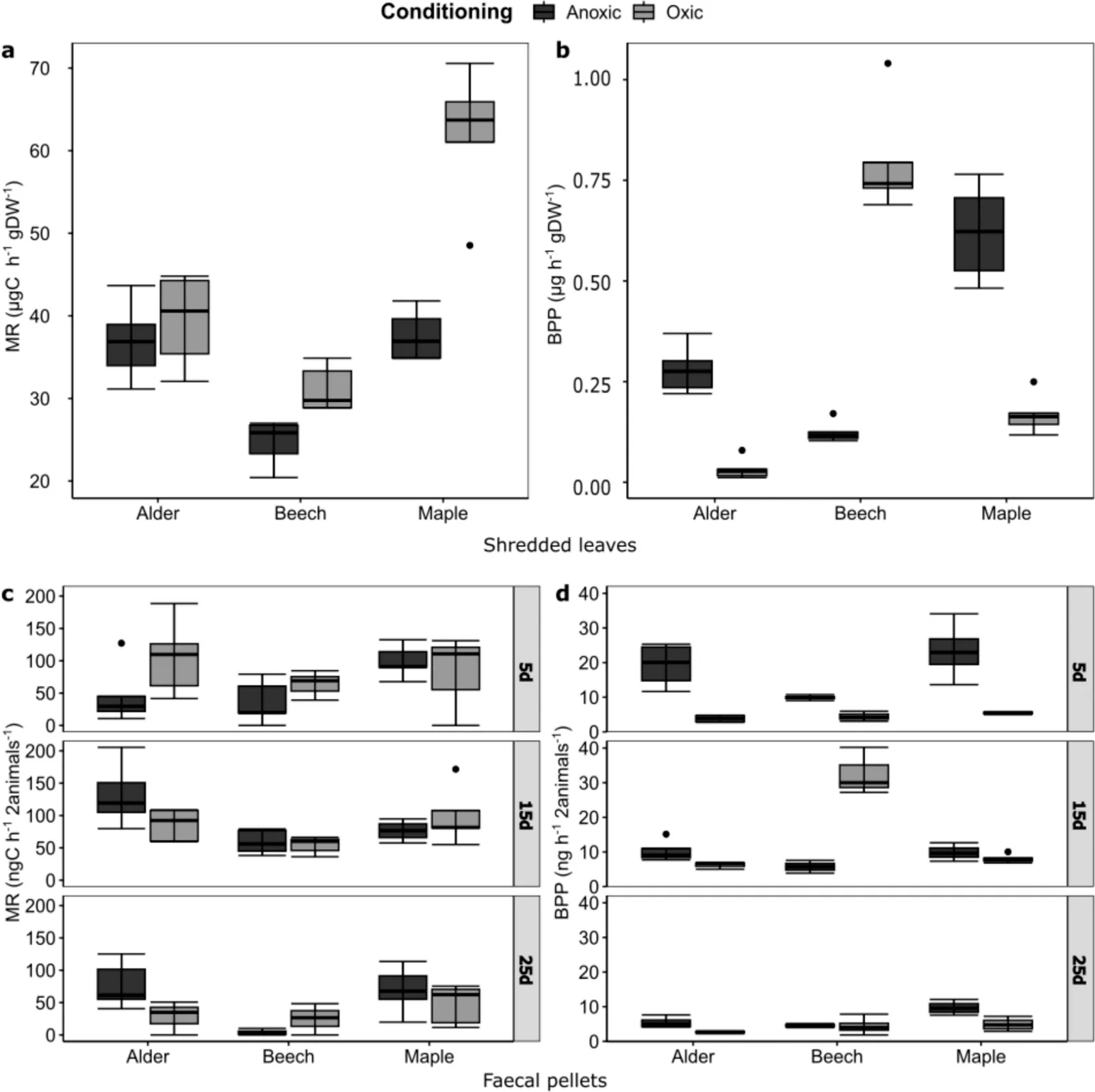
Microbial respiration (MR) (**a**) and bacterial protein production (BPP) (**b**) on shredded leaves at the start of the experiment. The microbial respiration (**c**) and bacterial protein production (**d**) on faecal pellets collected on the 5th day (5d), 15th day (15d) and 25th day (25d) of the feeding experiment. The two colours distinguish the samples from oxic or anoxic conditioning. The boxplots visualize the median of each treatment (line), the first and third quartiles (hinges) and the 1.5 × inter-quartile ranges (whiskers), and the dots indicate outside values greater than 1.5 × and less than 3 × the interquartile range

We observed a combined effect of leaf species and conditioning on both microbial respiration (MR) and bacterial protein production (BPP) on shredded leaves (Tables 2 and 3). Generally, MR was lower on anoxic-conditioned leaves whereas BPP was higher on anoxic-conditioned leaves, except for shredded beech leaves where we observed approximately six times higher BPP on the oxic-conditioned than anoxic-conditioned shredded leaves (Table S3). We measured the highest MR of 61.95 ± 8.28 µgC h^−1^ gDW^−1^ on oxic-conditioned shredded maple leaves (Fig. 3a; Table S3) and the lowest MR of 24.66 ± 2.78 µgC h^−1^ gDW^−1^ on anoxic-conditioned shredded beech leaves (Fig. 3a; Table S3). Furthermore, we found the highest BPP of 0.69 ± 0.12 µg h^−1^ gDW^−1^ on oxic-conditioned shredded beech leaves and the lowest BPP of 0.03 ± 0.02 µg h^−1^ gDW^−1^ on oxic-conditioned shredded alder leaves (Fig. 3b, Table S3).

**Table 2:** Effects of leaf species, conditioning and their interactions on MR of shredded leaves during feeding experiment based on linear model analysis. Abbreviations: St, standardized; SS, sum of squares; df, degrees of freedom

Factors	St. coefficient	SS	*df*	*F*-value	*p*-value
Conditioning	0.03	0.010	1	0.686	0.416
Leaf species	0.62	0.548	2	18.377	**<** **0.001**
Conditioning: Leaf species	0.39	0.219	2	12.268	**< 0.001**
Residuals		0.343	23	7.349	

Statistically significant *p*-values (< 0.05) are shown in bold

**Table 3:** Effect of leaf species, conditioning and their interactions on BPP of shredded leaves and faecal pellets collected on different sampling times during the feeding experiment based on linear model analysis. Abbreviations: St, standardized; SS, sum of squares; df, degrees of freedom

FPOM type	Time	Factors	St. coefficient	*SS*	*df*	*F*-value	*p*-value
Shredded leaves	Start	Conditioning	0.77	0.315	1	79.936	**< 0.001**
		Leaf species	0.83	0.474	2	60.112	**< 0.001**
		Conditioning: Leaf species	0.94	1.368	2	173.378	**< 0.001**
		Residuals		0.094	24		
Faecal pellets	5d	Conditioning	0.79	5.148	1	61.633	**< 0.001**
		Leaf species	0.52	1.441	2	8.627	**0.003**
		Conditioning: Leaf species	0.30	0.574	2	3.437	0.058
		Residuals		1.337	16		
	15d	Conditioning	0.31	0.391	1	6.587	**0.021**
		Leaf species	0.48	0.819	2	6.906	**0.007**
		Conditioning: Leaf species	0.85	5.052	2	42.609	**< 0.001**
		Residuals		0.889	15		
	25d	Conditioning	0.21	13.368	1	4.463	0.050
		Leaf species	0.54	60.486	2	10.098	**0.001**
		Conditioning: Leaf species	0.30	21.787	2	3.637	**0.048**
		Residuals		50.915	17		

Statistically significant *p*-values (< 0.05) are shown in bold

There was also a combined effect of leaf species, conditioning and time on MR on faecal pellets (Tables 4 and S5). Further, we found different MR and BPP on faecal pellets between the oxic and anoxic conditioning and leaf species on 5d (Tables 3, 4, S5, S6 and S7). BPP in 5d faecal pellets from shredders feeding on oxic-conditioned leaves was lower than from anoxic-conditioned leaves (Table S3). However, these differences were no longer evident in 15d and 25d faecal pellets with similar MR and BPP for most leaf species and conditioning, (Tables S3, S5, S6 and S7) and with microbial activity decreasing over time (Table S3).

**Table 4:** Model fit of a linear mixed model describing effects of leaf species, conditioning, time and their interactions on MR of faecal pellets. Abbreviations: St, standardized; SS, sum of squares; MS, mean of squares; df (Num; Den), degrees of freedom (nominator; denominator)

Factors	St. coefficient	SS	MS	*df* (Num;Den)	*F*-value	*p*-value
Conditioning	0.01	127.9	127.9	1;28.0	0.152	0.700
Leaf species	0.33	11,427.3	5713.6	2;27.9	6.779	**0.004**
Time	0.31	15,864.1	7932.0	2;41.3	9.411	**< 0.001**
Leaf species: Conditioning	0.04	965.2	482.6	2;27.9	0.573	0.571
Leaf species: Time	0.15	6056.9	1514.2	4;40.8	1.797	0.148
Conditioning: Time	0.17	7250.3	3625.1	2;41.2	4.301	**0.020**
Leaf species: Conditioning: Time	0.21	9036.5	2259.1	4;40.8	2.680	**0.045**

Statistically significant *p*-values (< 0.05) are shown in bold

**Fig. 4:**
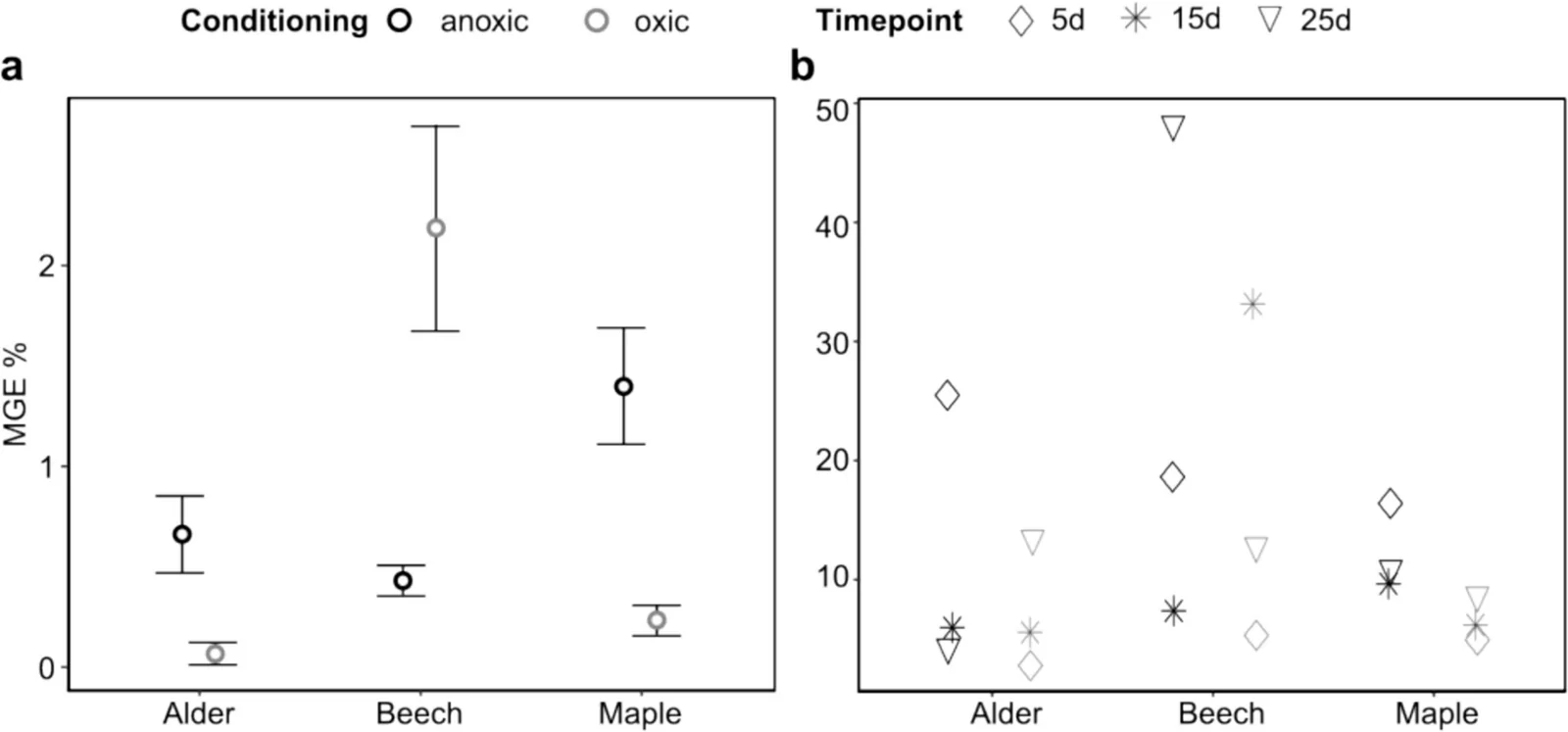
Microbial growth efficiency (MGE) on shredder-produced FPOM with leaves (mean and standard error; *n* = 29) (**a**) and faecal pellets (*n* = 18) at different timepoints (**b**). The black and grey lines distinguish the samples from anoxic or oxic conditioning. The three different symbols in **b** indicate three timepoints. Note the different scales of the y axes for **a** and **b**

We additionally calculated the microbial growth efficiency (MGE) from MR and BPP to be able to compare microbial activities on shredded leaves versus faecal pellets and test their differences. The MGE was significantly higher on faecal pellets (13.4 ± 12.1%) compared to shredded leaves at the start (0.81 ± 0.79%) (type III ANOVA; *F*_(1,35)_ = 4.97; *p* < 0.05; see Tables S8, S9 for full model output, Fig. 4). Additionally, we found an effect of leaf species on MGE of both leaves and faecal pellets (type III ANOVA; *F*_(2,35)_ = 3.41; *p* < 0.05; Fig. 4).

## Discussion

In the present study, we explored the nutritional composition and microbial activities of shredder-produced FPOM in response to leaf species and conditioning. Overall, our results indicate that CPOM properties, such as the C/N ratio, are partially reflected in shredder-produced FPOM. Conditioning enhanced the C/N ratios for low-quality CPOM. Yet, we neither found differences between oxic- or anoxic-conditioned leaves nor observed changes after digestion in shredder’s guts, in contrast to our hypotheses. Nevertheless, we detected clear differences between the two types of shredder-produced FPOM, i.e. shredded leaves and faecal pellets, regarding their FA composition and MGE. Our findings thus suggest that leaf conditioning and shredder activity shape FPOM chemical composition and activity of FPOM-associated microbial communities during leaf breakdown.

### CPOM Quality Is Mirrored in Shredder-Produced FPOM

We predicted that different conditions during microbial colonization during leaf conditioning would influence the C/N ratio of the leaves. However, we did not find differences between anoxic- and oxic-conditioned leaves and observed decreases in C/N ratios for low-quality CPOM prior to and after conditioning. Leaching and microbial colonization independent of oxygen availability during the conditioning thus may have altered the elemental composition of beech and maple leaves as shown in other publications [37, 38]. As C/N ratios of microbial biomass are generally lower (5 to 15 for bacteria [39]) than those of leaf litter [40, 41], it is plausible that microbial biofilms on CPOM reduced the overall C/N ratios on beech and maple leaves during the conditioning phase. Additionally, the increase in the C/N ratio of beech leaves during the feeding experiment could potentially be attributed to the selective feeding preference of shredders on microbe-colonized leaf areas, as suggested by Fenoy et al. [42] and Graça [9].

CPOM-associated biofilms are considered the primary energy and nutrient resource for invertebrate shredders [8, 43], while leaves themselves are of low nutritional value [37]. Further, it is commonly assumed that the shredder activity, encompassing both shredding behaviour along with feeding preference, and gut passage, produces FPOM with higher C/N ratios by removing N from leaf particles [9, 42, 44, 45]. However, our findings on faecal pellets challenge this concept, as we observed similar C/N ratios on CPOM and faecal pellets pointing towards no selective assimilation of certain nutrients, such as N, during gut passage. Hence, we can assume that the CPOM quality is indeed mirrored in shredder-produced FPOM. As microbes colonizing the leaves may influence the C/N ratio of the food resource for shredders [5, 25], we support the assumption that microbial colonization drives the nutritional value for aquatic consumers [8, 43] but at the same time, our results indicate that shredders do not necessarily produce low quality or nutrient-poor FPOM [44, 45].

### Activity of FPOM-Associated Microbial Communities Is Mostly Influenced by Conditioning

The findings of our study uncovered that different leaf species and conditioning had significant effects on the FPOM-associated microbial metabolic processes. Additionally, leaf species alone significantly influenced the microbial activities associated with shredded leaves and faecal pellets during the feeding experiment. This may be directed towards the influence of varying nutritional value of leaf species on the microbial community composition associated with shredder-produced FPOM and subsequently, the activity of microorganisms, as evidenced by previous studies [26, 46]. Furthermore, we found, compared to the anoxic ones, significantly higher MR on oxic-conditioned shredded leaves and faecal pellets collected by feeding oxic-conditioned leaves on 5d. Anoxic conditioning selects for anaerobes as members of the active microbial community, and anaerobic processes are typically slower in carbon turnover during the conditioning phase that was still persisting during the subsequent oxic experimental phase [47, 48]. The switch from an anoxic to oxic conditioning phase might have resulted in the availability of incompletely degraded organic matter that could now be used efficiently for growth by obligatory and facultative aerobes under oxic conditions. Moreover, the minimal turnover of primary fermentation products [49] during anoxic conditions and the revival of fungi that were inhibited under anoxic conditions [50] could have further boosted the growth of the microbial community. This could be the argument behind the higher BPP on anoxic-conditioned alder, maple leaves and faecal pellets collected on 5d. Yet, we did not perform any molecular techniques to validate the revival of fungi. Nevertheless, the observed pattern of a higher oxic MR and lower oxic BPP still holds true for the faecal pellets collected at the beginning of the experiment, indicating that the microbial metabolic processes on the produced FPOM might be preserved even after gut passage.

The distinct effect of conditioning on faecal pellet associated microbial activity with a higher MR on oxic-conditioned but higher BPP on faecal pellets obtained by feeding previously anoxic-conditioned leaves disappeared with time. This is probably attributed to a shift in activity and composition of the microbial communities on the formerly anoxic-conditioned leaves now maintained under oxic conditions during the feeding experiment. Yet, the effect of different leaf species was significant for both MR and BPP in faecal pellets over time, suggesting other factors could also play a role (e.g. organic matter quantity or quality) [25, 51]. Additionally, the senescence of the leaves over time may also contribute to a decrease in microbial activities, as the resources and quality of the leaves that the shredders were feeding on decrease in parallel. This is supported by our observation of increased C/N ratios in beech leaves from the start to the end of the feeding experiment. Hence, the large variability in the microbial metabolism observed in the shredder-produced FPOM in our experiment emphasizes the notable effects of the preceding environmental conditions during the leaf conditioning and the shredder activity in determining microbial community dynamics during LLD in streams.

### Gut Passage Influences the Composition and Processing of Shredder-Produced FPOM

Our findings, based on fatty acid composition and MGE, revealed a clear difference between shredded leaves and faecal pellets, indicating differential composition and processing of organic matter by shredders and associated microbes. These differences were much more pronounced than those observed among leaf species and conditioning. However, if this finding would hold true for faecal pellets produced by different shredder taxa remains to be investigated. Further, we showed that the MGE of the faecal pellets was markedly higher than that of the shredded leaves pointing towards competitive interactions between microbial communities on leaves [52] and the gut passage effect on faecal pellets with bacterial dominance [53]. This speculation is supported by our FA data showing significantly higher bacterial biomarkers on the faecal pellets, possibly indicating a relatively greater role of bacteria on this type of shredder-produced FPOM compared to fungi or other eukaryotic microorganisms such as algae, which may be more important on the leaves.

Leaf species influence the leaf-associated microbial community, which consists of eukaryotic and prokaryotic microorganisms as described in previous LLD studies [4, 54, 55]. It is noteworthy that our study was the first to investigate the activity and FA composition of microbial communities on shredder-produced faecal pellets from *Sericostoma* sp. after feeding on different leaf species. Additionally, our data showed that the microbial community on faecal pellets has a dominance of bacteria that apparently are very efficient in utilizing this carbon source, as evidenced by their relatively high MGEs [34]. From previous studies, it is well known that n-3 PUFAs as algal biomarkers are a high-quality food source for aquatic consumers, particularly in freshwater ecosystems [27]. Furthermore, our data showed that most of the bulk n-3 PUFA consisted of short-chain n-3 PUFA (ALA) (Table S10), which can be synthesized by both fungi and green algae. However, we did not use any other specific technique to verify whether the source of short-chain n-3 PUFA in our study was from fungi or exclusively from algae. Nevertheless, we speculate that both algal and fungal biofilms growing on leaf surfaces (Table S10) can enhance the nutritional quality of food for consumers [5, 27, 56], support their somatic growth [6] and fulfill their physiological requirements [56]. Consequently, it is possible that certain fatty acids are selectively retained during gut passage and/or others preferentially degraded [56, 57]. This can be the explanation for the higher amounts of n-3 PUFA on shredded leaves compared to faecal pellets. Therefore, this result suggests that gut passage enhances the colonization of bacteria and eventually promotes the growth of heterotrophic microbial communities in faecal FPOM.

## Conclusions

Microorganisms, including heterotrophic bacteria and aquatic hyphomycetes, can affect LLD in various ways, including the colonization and decomposition of leaves and faecal pellets for aquatic organisms such as shredders, collectors and gatherers. However, the role and activities of microorganisms on shredder-produced FPOM have been understudied so far. We showed that microbial colonization on CPOM at two oxygen levels altered the resource quality and, consequently, the microbial metabolism on the shredded leaves and faecal pellets. Our results show that faecal pellets provide good conditions for microbial communities to thrive, underscoring that faecal pellets as microbial hotspots might play a key role in accelerating nutrient cycling in aquatic ecosystems. Our study illustrates how detritivore-microbe interactions influence ecosystem functions, thus contributing to the understanding of the complexity of organic matter decomposition and how environmental and biological factors interact to shape ecosystem processes. Our results call for more studies on the identity and structure of FPOM-associated microbial communities, using advanced molecular techniques like metagenomics or meta-transcriptomics. This will further deepen our knowledge about the key microbial communities and their impact on the FPOM nutritional value along with their metabolic functions and interactions with shredders, thus advancing our understanding of FPOM dynamics in freshwater ecosystems.

## Supplementary Information

Below is the link to the electronic supplementary material.

Supplementary file1 (PDF 440 KB)

## Data Availability

The data presented in this study are available on request from the corresponding author.
