# Supplementary material for: Leaf Conditioning and Shredder Activity Shape Microbial Dynamics on Fine Particulate Organic Matter Produced During Decomposition of Different Leaf Litter in Streams
Source: Microb Ecol. 2025 Mar 22;88(1):18. doi: 10.1007/s00248-025-02515-2 (PMC11928410; doi:10.1007/s00248-025-02515-2)
Supplement: Supplementary file 1 — Supplementary file1 (PDF 440 KB) [file 248_2025_2515_MOESM1_ESM.pdf]

Supplementary Information (SI)

Leaf conditioning and shredder activity shape microbial dynamics on fine particulate organic matter produced during decomposition of different leaf litter in streams

Pratiksha Acharya[1,2], Mourine J. Yegon[1,3], Leonie Haferkemper[1,4], Benjamin Misteli[1], Christian Griebler[2], Simon Vitecek[1,3,5], Katrin Attermeyer*[1,2]

[1] WasserCluster Lunz – Biological Station, Dr. Carl Kupelwieser-Prom. 5, 3293 Lunz am See, Austria

[2] Department of Functional and Evolutionary Ecology, Unit Limnology, University of Vienna, Djerassiplatz 1, 1030 Vienna, Austria

[3] Institute for Hydrobiology and Water Management (IHG), University of Natural Resources and Life Sciences, Gregor-Mendel-Straße 33/DG, 1180 Vienna, Austria

[4] Department of Aquatic Ecology, Eawag, Swiss Federal Institute of Aquatic Science and Technology Überlandstrasse 133, 8600 Dübendorf, Switzerland

[5] Department of Ecology, University of Innsbruck, Technikerstraße 25, 6020 Innsbruck, Austria

*Correspondence: Katrin.Attermeyer@univie.ac.at**

Materials and Methods

Table S1. POC and PN concentrations of different leaf species used in this study. Data show mean ± standard deviation and the sample size for each analysis was n = 3.

**Leaf species**	**C (mol g^{1})**	**N (mol g^{1})**	**Molar C/N**
Alder	34.91±0.74	1.82±0.02	19.12±0.27
Beech	29.64±0.18	0.51±0.03	58.92±3.37
Maple	33.87±0.54	0.62±0.04	54.60±2.26

<u>Detailed description of experimental setup</u>

The feeding experiment was conducted in microcosms in a climate chamber at 12°C and a light:dark regime of 11:13 hrs. The experiment lasted for a total of 25 days (14th October - 7th November 2022). We set up six treatments with three leaf species conditioned under either oxic or anoxic conditions (i.e., Alder_anoxic, Alder_oxic, Beech_anoxic, Beech_oxic, Maple_anoxic, Maple_oxic) with five replicates resulting in 30 microcosms. Each microcosm consisted of a food-grade white plastic bucket with a volume of 1100 ml and a bottom diameter of 122 mm, in which we installed a steel mesh (pore size 1 mm) at a height of 4.5-5 cm from the bottom to allow the FPOM produced during leaf processing to pass through. We supplied 3.0 ± 0.2 g muffled sand particles (0.7-1.2 mm size) in aluminium petri-dishes to each microcosm for the shredders to build their cases. We filled each microcosm with 750 ml of stream water, which was bubbled with an aquarium pump connected to a tube and bubbling stone to sustain aerobic conditions throughout the whole experimental phase. We collected fresh stream water every four days to exchange the water in the microcosms. The water quality parameters dissolved organic carbon (DOC), nitrate (NO_3^-), nitrite (NO_2^-), ammonium (NH_4^+), and phosphorous (soluble reactive phosphorous = SRP) of the stream water were analysed before refilling the microcosms upon removal every fifth day.

We added ten shredders to each microcosm and fed them with the different conditioned leaves of one of the three species. After feeding for four days, each larva was transferred to a single food-grade plastic cup (Polylactic acid or polylactide (PLA), volume: 4 cl) filled with 20 ml of filtered stream water (0.7 µm, 450°C for 4 hrs pre-combusted Whatmann GF/F filters) and allowed to empty its gut for 24 hrs for faecal pellet collection. During the 24-hour period, the mesh and leaves were taken out and the water in the microcosms was replaced by freshly collected stream water to prevent the leaves from drying out. On the next day after 24 hrs, the faecal pellets in each cup were collected using a Pasteur pipette after the larvae were returned to their original microcosms. During the 25-day feeding experiment, we collected and analysed the larvae's faecal pellets three times: on the fifth, 15th, and 25th day (5d, 15d, and 25d). We collected the faecal pellets from each larva separately but pooled pellets from two larvae to have enough material for our measurements, providing us with five samples per microcosm and sampling. We measured bacterial protein production (BPP) and microbial respiration (MR) immediately, while samples for particulate organic carbon (POC) and particulate nitrogen (PN) content, and fatty acids (FA) were stored at -80 °C until further processing. At the end of the experiment, we additionally collected the leaf discs not consumed by the shredders and stored them at -80°C until further processing.

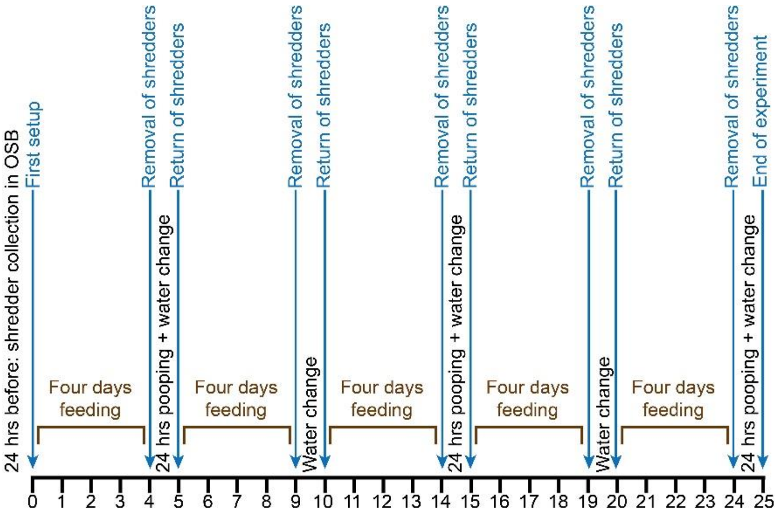

Figure S1. Timeline of the 25d-experiment with the references to the days when faecal pellets were collected.

Physio-chemical characterization of stream water

We characterised the water quality of the stream water before refilling the microcosms and upon removal after four days by measuring different physical and chemical water parameters. We measured pH using a pH meter (WTW pH 3310; Xylem Analytics, Weilheim, Germany) and conductivity using a conductivity meter (WTW Cond 3310; Xylem Analytics, Weilheim, Germany). Water temperature and dissolved oxygen concentrations were measured using an Oximeter (WTW Oxi 315i; Xylem Analytics, Weilheim, Germany). The concentrations of dissolved nutrients (ammonium, NH_4-N; nitrate, NO_3-N, nitrite, NO_2-N, and soluble reactive phosphorus, SRP) were measured using a continuous flow nutrient analyser (CFA, Alliance Instruments GmbH, Salzburg, Austria). We also analysed the DOC concentrations on a total organic carbon (TOC) analyser (Shimadzu Corporation, Kyoto, Japan) through combustion catalytic oxidation of the sample and NDIR-detection.

Table S2. Physical and chemical characteristics of the sampled water from the stream and six microcosm treatments: water temperature (T), pH, dissolved oxygen (dO), specific electrical conductivity (Cond.), and dissolved organic carbon (DOC). Dissolved nutrient concentrations (ammonium, N-NH_4, nitrate, N-NO_3, and soluble reactive phosphorus, SRP) are shown as mean values ± standard deviation (SD). Physical parameters were measured for each microcosm for each water replacement i.e., n = 25, while chemical parameters were measured in one microcosm from each treatment for each water replacement i.e., n = 5.

Samples	T [°C]	pH	dO [mg/L]	Cond. [µS/cm]	DOC [mg/L]	N-NH_4 [µg/L]	N-NO_2 [µg/L]	N-NO_3 [µg/L]	SRP [µg/L]
Stream	11.9±0.1	8.3±0.1	9.8±1.7	257.6±3.7	2.2 ± 0.4	4.1±2.2	2.4±0.4	584±64	0.84±0.5
Alder_anoxic	11±0.8	8.3±0.1	11.9±2.4	281±18	4.6±0.8	287±148	11.9±2.2	606±70	0.33±0.12
Beech_anoxic	11.1±0.4	8.2±0.1	11.8±2.2	267±8	3.1±0.8	104±94	8.2±3.5	564±59	19.1±18. 6
Maple_anoxic	10.4±0.8	8.3±0.1	12.2±2.2	279±22	4.17±0.3	33±26	12.1±2.3	474±78	0.53±0.12
Alder_oxic	11.1±0.3	8.3±0.1	12.1±2.7	277±16	6.6±2.3	426±183	11.4±3.5	516±90	0.5±0.2
Beech_oxic	10.5±0.3	8.3±0.1	12.8±2.9	276±15	3.5±0.5	64.74±104	6.2±1.6	518±56	5.4±8.5
Maple_oxic	10.9±0.3	8.2±0.1	12.1±3.1	267±6	5.2±0.9	70±58	8.1±1.9	349±120	1.65±0.94

FPOM elemental and fatty acid composition

For elemental and fatty acid composition analyses, the amount of faecal pellets from two larvae per microcosms were insufficient. Hence, we pooled the five samples per treatment (faecal pellets per 2 larvae *5 microcosm per treatment = faecal pellets from 10 larvae), which gives us only one sample per treatment.

For the analyses of the elements organic C and N, we first freeze dried (Virtis™ Genesis Freeze Dryer) the collected leaf samples from start and end, and faecal pellets from different time points for 2 days. For POC and PN analyses, we grounded and homogenized all samples and weighed about 1-1.2 mg into tin capsules (5 mm x 9 mm; IVA Analysentechnik GmbH & Co. KG, Meerbusch, Germany) using a microbalance (Sartorius CPA2P, Sartorius Lab Instruments GmbH & Co. KG, Göttingen, Germany). For determining the POC and PN content of the samples, we used Algae standards (Bladderwrack; 1-1.2 mg DW, IVA Analysentechnik GmbH & Co. KG, Meerbusch, Germany). We measured the elemental composition of all prepared samples on a Flash HT Plus

CHNS/O elemental analyser (Thermo Fisher Scientific, Bremen, Germany) and the C/N ratios reported here are expressed as molar ratios.

We conducted FA analysis following the procedures described in Guo et al. (2016) as a three-step process consisting of lipid extraction, derivatisation to fatty acid methyl esters (FAMEs), and quantification of FAMEs using gas chromatography (GC). Briefly, we mixed freeze-dried samples with chloroform:methanol (2:1 V/V) following the addition of 0.9% NaCl, sonication, vortexing, and centrifuging three times to remove non-lipid materials. We evaporated pooled organic phases to a final volume of 1.5 mL under N_2 gas flow. For FAME formation, we incubated a known volume of lipid extracts with sulfuric acid:methanol (1:100 V/V) for 16 h at 50°C, followed by the addition of toluene, $KHCO_3$, and hexane. Samples were shaken, vortexed, CO_2 released and centrifuged for three minutes at 4°C and 1500 rpm, and the upper organic layers were collected twice, pooled, and concentrated under N_2 to obtain a defined volume for the analysis. We quantified FAME on a gas chromatograph (Trace GC with TriPlus RHS Autosampler, Thermo Fisher Scientific, Waltham, MA, USA, Detector: FID 260 °C, Carrier gas: He: 1 mL/min, Detector gases: H_2: 40 mL/min, N_2: 45 mL/min, air: 450 mL/min) with an HP-88 column (length: 100 m, inner diameter: 0.25 mm, film thickness: 0.2 µm, format: 7 inch; Agilent Technologies, Inc., Santa Clara, CA, USA) for FAME separation. FAMEs were identified by comparing their retention time with known reference standards (Supelco 37 Component FAME Mix, CRM47885; and Bacterial Acid Methyl Ester (BAME) Mix, 47080-U; both obtained from Sigma-Aldrich, Merck KGaA, Darmstadt, Germany) and two in-house standards for stearidonic acid (18:4n-3) and eicosatetraenoic acid (20:4n-3). In total, these standards allowed to identify 47 single FAs. We used the software Chromeleon 7™ (Thermo Fisher Scientific, Waltham, MA, USA) for manual peak adjustment and calculated concentrations of FA based on individual calibration curves. Results were expressed as mass fractions (µg FAME/mg DW) and reported as mass percentages (% total FAME).

<u>FPOM-associated microbial activities</u>

At the start of the feeding experiment, we finely chopped five to six conditioned leaf discs from each leaf species (= 6 samples*5 replicates) with a knife. This process was mimicking the sloppy feeding of the shredders when eating the leaves and we refer to this type of FPOM as “shredded leaves” throughout the manuscript. We immediately incubated a known amount of shredded leaves for measurements of MR and BPP. In addition, we incubated the faecal pellets pooled from two larvae per microcosm on the fifth, 15th, and 25th day (5d, 15d, and 25d) of the feeding experiment for MR and BPP measurements.

We quantified respiration via oxygen consumption over time with a needle-type O_2 microsensor (Microx 4, Presens GmbH, Regensburg, Germany). We weighed about 0.099 ± 0.074 g of shredded leaves into 10 mL precombusted glass vials and filled each vial to the rim with filtered stream water (0.7 µm, precombusted Whatmann GF/F filters). We closed the vials with a lid with butyl rubber septum through which the O_2 microsensor was inserted. For each treatment, we filled five vials with the shredded leaves (= 30 samples). We also incubated five vials filled with filtered stream water as blank. For faecal pellets, we transferred the faecal pellets pooled from two larvae into 10-ml precombusted glass vials and filled the vials with the stream water from the plastic cups after collection of the faecal pellets. Additionally, we incubated the water from the plastic cups without faecal pellets as blank. For each treatment, we had five replicates (= 30 samples + 5 blanks). All samples were incubated for 72 hrs at in situ temperatures in the dark and O_2 concentrations were measured at regular intervals (every 12 hrs). Before each

measurement, the vials were carefully mixed by turning them upside down. We calculated MR from linear regressions of oxygen depletion over time. We converted the amount of consumed O_2 to µg C L^{-1} d^{-1} by using a conversion factor of 1 (Berggren et al., 2012). We here express MR for shredded leaves and faecal pellets as µg C h^{-1} gDW^{-1} and ng C h^{-1} 2 $animals^{-1}$, respectively. We converted the MR values from L to gDW by determining the dry weight of the weighed wet leaves after the MR measurement for each replicate. To do this, we dried the leaves at 60°C for 48 hours.

We determined BPP via incorporation of L-Leucine [4,5-3H] into the protein fraction using the protocol of Simon and Azam (1989) and Buesing and Gessner (2003). We incubated a volume of 1 mL for FPOM types in 2 mL Eppendorf tubes. For shredded leaves, it was 0.058 ± 0.018 g of shredded leaves with 1 mL filtered stream water (0.22 µm cellulose acetate syringe filters (VWR)). For faecal pellets, it was 0.5 mL faecal pellets as we always collected small amounts of water when picking the faecal pellets, which was mixed with 0.5 mL filtered stream water. We added 32 µL working solution of leucine (Hartmann Analytic, Braunschweig, Germany, specific activity 39.90 Ci $mmol^{-1}$) at a final concentration of 125 nmol L^{-1}. Prior to incubation, we added 50 µL 100% trichloroacetic acid (TCA) (5% final concentration) to the respective blanks and at the end of the incubation after one hour to each sample. All incubations were run in four replicates and one blank per treatment. For optimal coagulation of the proteins after TCA addition, we left the samples for 10 min at room temperature in the dark before centrifugation. After centrifugation at 14,000 rpm (15,800 x g) for 10 min, we decanted the supernatant and washed the pellets with 1 mL ice-cold 5% TCA. We washed the faecal pellets again with 1 mL ice-cold 80% ethanol, each time alternating with a vigorous vortexing and 10-min centrifugation. Afterwards, we added 1 mL scintillation cocktail (Ultima Gold; PerkinElmer, Inc., Waltham, MA, USA) and let the samples sit overnight. The next day, we counted disintegrations per minute (DPM) on a liquid scintillation counter (LS6500; Beckman Coulter, Brea, CA, USA). Net disintegrations per minute were converted to pmol leucine L^{-1} d^{-1} according to Simon and Azam (1989), and an isotope dilution factor of 2 was applied (Kirchman, 1993). We report BPP as µg h^{-1} gDW^{-1} for shredded leaves and ng h^{-1} 2 $animals^{-1}$ for faecal pellet samples.

Due to some negative numbers after blank subtraction for MR and BPP measurements on faecal pellets, we removed 22 samples (15 MR + 7 BPP) from the data analysis leading to unequal observations/replication as for each treatment (this is referred to as an unbalanced dataset here). This issue may have emerged due to insufficient amounts of faecal pellets obtained from two larvae. Small sample size, therefore, confined us to run just a linear model instead of a linear mixed model for BPP in faecal pellets. Afterwards we evaluated the model fit by running a Type III ANOVA (function *Anova*; *car* package) and reported the significant factors after calculating the estimated marginal means and the upper and lower limits of the 95% confidence interval (*emmeans* package) and applying multivariate t-distribution adjustment approach for pairwise comparisons among the groups.

Table S3. MR and BPP measured in leaves (MR; µg C h^{-1} gDW^{-1}, BPP; µg h^{-1} gDW^{-1}) at start and in faecal pellets (MR; ng C h^{-1} 2 $animals^{-1}$, BPP; ng h^{-1} 2 $animals^{-1}$) at different timepoints during 25 days of feeding experiment. Median, mean, and standard deviation (SD) are shown.

				**MR**			**BPP**		
FPOM type	Time	Leaf species	Conditioning	Mean	Median	SD	Mean	Median	SD
Leaves	start	Alder	Anoxic	36.93	36.88	4.79	0.246	0.28	0.05
		Beech	Anoxic	24.66	25.85	2.79	0.112	0.13	0.02
		Maple	Anoxic	30.10	34.93	3.37	0.53	0.62	0.10
		Alder	Oxic	39.43	40.59	5.57	0.03	0.33	0.02
		Beech	Oxic	31.14	29.6	2.78	0.69	0.79	0.12
		Maple	Oxic	61.95	63.71	8.28	0.156	0.17	0.04
Faecal pellets	5d	Alder	Anoxic	46.79	29.48	41.69	20.04	19.26	5.72
		Beech	Anoxic	35.86	19.82	29.42	9.87	9.88	0.61
		Maple	Anoxic	98.99	90.87	22.25	22.93	23.39	7.32
		Alder	Oxic	105.46	109.72	51.70	3.83	3.80	0.92
		Beech	Oxic	64.28	69.12	16.21	4.28	4.43	1.18
		Maple	Oxic	80.56	110.68	57.56	5.52	5.44	0.35
	15d	Alder	Anoxic	132.05	119.38	43.29	9.06	10.25	2.90
		Beech	Anoxic	59.06	56.07	16.54	5.72	5.72	1.39
		Maple	Anoxic	76.37	76.61	14.32	9.64	9.88	2.18
		Alder	Oxic	85.74	92.32	21.87	6.73	6.19	0.82
		Beech	Oxic	54.33	59.93	11.41	30.01	32.47	5.59
		Maple	Oxic	99.37	81.68	39.76	7.60	8.02	1.21
	25d	Alder	Anoxic	76.75	61.38	31.53	4.96	5.43	1.41
		Beech	Anoxic	4.06	1.93	4.40	4.56	4.55	0.43
		Maple	Anoxic	69.50	67.67	31.94	9.56	9.69	1.74
		Alder	Oxic	24.97	26.58	19.76	2.75	2.64	0.31
		Beech	Oxic	47.753	62.35	26.98	4.01	4.43	2.17
		Maple	Oxic	17.24	1.45	23.36	4.76	4.91	1.66

Table S4. Result of the linear model to observe the effects of leaf species, conditioning, and their combined effects on MR of shredded leaves. The model structure is MR ~ leaf species*conditioning. Abbreviations; SE: Standard error.

**Factors**	***Estimate***	***SE***	***t-value***	***p-value***
ConditioningOxic	0.064	0.077	0.828	0.415
Leaf speciesBeech	-0.402	0.077	-5.211	**< 0.001**
Leaf speciesMaple	0.022	0.082	0.275	0.785
ConditioningOxic:Leaf speciesBeech	0.171	0.109	1.571	0.130
ConditioningOxic:Leaf speciesMaple	0.429	0.113	3.820	**< 0.001**

Statistically significant p-values (< 0.05) values are shown in bold

Table S5. Results of the linear mixed model for microbial respiration (MR) measured on faecal pellets collected at different sampling times during the feeding experiment. Leaf species, conditioning and time were included as fixed factors, and Microcosms ID as the random factor. The model structure is MR ~ leaf species*conditioning*time, (1 | mesocosm ID). Abbreviations; SE: Standard error, df: degrees of freedom.

**Fixed effects**	*Estimate*	*SE*	*df*	*t-value*	*p-value*
Leaf speciesBeech	-72.983	21.133	54	-3.453	**0.001**
Leaf speciesMaple	-53.910	22.295	55	-2.418	**0.019**
ConditioningOxic	-46.303	21.133	54	-2.191	**0.033**
time25d	-55.293	18.361	36	-3.011	**0.005**
time5d	-85.260	18.361	36	-4.643	**<0.001**
Leaf speciesBeech:ConditioningOxic	41.567	29.887	54	1.391	0.170
Leaf speciesMaple:ConditioningOxic	67.540	30.719	55	2.199	**0.032**
Leaf speciesBeech:time25d	8.561	31.327	42	0.273	0.786
Leaf speciesMaple:time25d	46.660	26.920	38	1.733	0.091
Leaf speciesBeech:time5d	69.891	26.934	38	2.595	**0.013**
Leaf speciesMaple:time5d	106.110	26.920	38	3.942	**<0.001**
ConditioningOxic:time25d	3.961	31.256	41	0.127	0.900
ConditioningOxic:time5d	104.980	25.967	36	4.043	**<0.001**
Leaf speciesBeech:ConditioningOxic:time25d	27.416	47.549	43	0.577	0.567
Leaf speciesMaple:ConditioningOxic:time25d	-46.947	41.251	40	-1.138	0.262
Leaf speciesBeech:ConditioningOxic:time5d	-79.654	37.413	37	-2.129	**0.040**
Leaf speciesMaple:ConditioningOxic:time5d	-104.998	41.251	40	-2.545	**0.015**
**Random effects**	Variance	SD			
Microcosms_ID	273.7	16.54			
Residual	842.8	29.03			

Statistically significant p-values (< 0.05) values are shown in bold

Table S6. Result of the linear model to observe the effects of leaf species, conditioning, and their interactions in BPP of shredded leaves and faecal pellets collected at different sampling times during the feeding experiment. The model structure is BPP ~ leaf species*conditioning. Abbreviations; SE: Standard error.

FPOM type	Time	Factors	*Estimate*	*SE*	*t-value*	*p-value*
Shredded leaves	start	ConditioningOxic	-0.355	0.040	-8.941	**<0.001**
		Leaf speciesBeech	-0.176	0.040	-4.419	**<0.001**
		Leaf speciesMaple	0.257	0.040	6.481	**<0.001**
		ConditioningOxic:Leaf speciesBeech	0.895	0.056	15.931	**<0.001**
		ConditioningOxic:Leaf speciesMaple	-0.022	0.056	-0.384	0.704
Faecal pellets	5d	ConditioningOxic	-1.604	0.204	-7.851	**<0.001**
		Leaf speciesBeech	-0.621	0.204	-3.037	**0.008**
		Leaf speciesMaple	0.191	0.204	0.936	0.363
		ConditioningOxic:Leaf speciesBeech	0.768	0.301	2.554	**0.021**
		ConditioningOxic:Leaf speciesMaple	0.197	0.301	0.653	0.523
	15d	ConditioningOxic	-0.477	0.186	-2.567	**0.021**
		Leaf speciesBeech	-0.578	0.172	-3.357	**0.004**
		Leaf speciesMaple	-0.026	0.186	-0.140	0.890
		ConditioningOxic:Leaf speciesBeech	2.230	0.263	8.478	**<0.001**
		ConditioningOxic:Leaf speciesMaple	0.283	0.263	1.077	0.299
	25d	ConditioningOxic	-2.793	1.322	-2.113	0.05
		Leaf speciesBeech	-0.874	1.224	-0.714	0.485
		Leaf speciesMaple	4.265	1.224	3.485	**0.003**
		ConditioningOxic:Leaf speciesBeech	2.665	1.801	1.48	0.157
		ConditioningOxic:Leaf speciesMaple	-1.988	1.801	-1.103	0.285

Statistically significant p-values (< 0.05) values are shown in bold

Table S7. Result of the contrast test to observe pairwise comparisons among the groups based on marginal mean values of MR and BPP. The p-values were obtained after applying multivariate *t*-distribution adjustment. Abbreviations; SE: Standard error, df: degrees of freedom.

			**MR**					**BPP**				
FPOM type	Time	Contrast	*Estimate*	*SE*	*df*	*t-value*	*p-value*	*Estimate*	*SE*	*df*	*t-value*	*p-value*
Shredded leaves	start	Alder Anoxic - Alder Oxic	-0.064	0.077	23	-0.828	0.959	0.355	0.040	24	8.941	**<0.001**
		Alder Anoxic - Beech Anoxic	0.402	0.077	23	5.211	**<0.001**	0.176	0.040	24	4.419	**0.002**
		Alder Anoxic - Beech Oxic	0.167	0.077	23	2.161	0.293	-0.364	0.040	24	-9.170	**<0.001**
		Alder Anoxic - Maple Anoxic	-0.023	0.082	23	-0.275	1.000	-0.257	0.040	24	-6.481	**<0.001**
		Alder Anoxic - Maple Oxic	-0.516	0.077	23	-6.689	**<0.001**	0.119	0.040	24	3.003	0.061
		Alder Oxic - Beech Oxic	0.231	0.077	23	2.989	0.063	-0.720	0.040	24	-18.111	**<0.001**
		Alder Oxic - Maple Oxic	-0.452	0.077	23	-5.861	**<0.001**	-0.236	0.040	24	-5.938	**<0.001**
		Beech Anoxic - Alder Oxic	-0.466	0.077	23	-6.040	**<0.001**	0.180	0.040	24	4.522	**0.002**
		Beech Anoxic - Beech Oxic	-0.236	0.077	23	-3.051	0.056	-0.540	0.040	24	-13.589	**<0.001**
		Beech Anoxic - Maple Anoxic	-0.425	0.082	23	-5.188	**<0.001**	-0.433	0.040	24	-10.900	**<0.001**
		Beech Anoxic - Maple Oxic	-0.919	0.077	23	-11.901	**<0.001**	-0.056	0.040	24	-1.416	0.718
		Beech Oxic - Maple Oxic	-0.683	0.077	23	-8.850	**<0.001**	0.484	0.040	24	12.173	**<0.001**
		Maple Anoxic - Alder Oxic	-0.041	0.082	23	-0.506	0.995	0.613	0.040	24	15.422	**<0.001**
		Maple Anoxic - Beech Oxic	0.189	0.082	23	2.312	0.229	-0.107	0.040	24	-2.689	0.115
		Maple Anoxic - Maple Oxic	-0.494	0.082	23	-6.032	**<0.001**	0.377	0.040	24	9.484	**<0.001**
Faecal pellets	5d	Alder Anoxic - Alder Oxic	-58.677	21.133	53	-2.776	0.344	1.604	0.204	16	7.851	**<0.001**
		Alder Anoxic - Beech Anoxic	3.093	22.377	55	0.138	1	0.621	0.204	16	3.037	0.071
		Alder Anoxic - Beech Oxic	-17.497	21.133	53	-0.828	1	1.457	0.221	16	6.600	**<0.001**
		Alder Anoxic - Maple Anoxic	-52.2	21.133	53	-2.47	0.545	-0.191	0.204	16	-0.936	0.931
		Alder Anoxic - Maple Oxic	-73.419	27.689	58	-2.652	0.42	1.217	0.221	16	5.512	**0.001**
		Alder Oxic - Beech Oxic	41.18	21.133	53	1.949	0.87	-0.148	0.221	16	-0.669	0.983
		Alder Oxic - Maple Oxic	-14.742	27.689	58	-0.532	1	-0.388	0.221	16	-1.757	0.517
		Beech Anoxic - Alder Oxic	-61.769	22.377	55	-2.76	0.352	0.984	0.204	16	4.814	**0.002**
		Beech Anoxic - Beech Oxic	-20.589	22.377	55	-0.92	1	0.836	0.221	16	3.788	**0.017**

	Beech Anoxic - Maple Anoxic	-55.293	22.377	55	-2.471	0.546	-0.812	0.204	16	-3.973	**0.012**
	Beech Anoxic - Maple Oxic	-76.511	28.649	58	-2.671	0.408	0.596	0.221	16	2.700	0.129
	Beech Oxic - Maple Oxic	-55.922	27.689	58	-2.02	0.836	-0.240	0.236	16	-1.018	0.905
	Maple Anoxic - Alder Oxic	-6.477	21.133	53	-0.306	1	1.796	0.204	16	8.786	**<0.001**
	Maple Anoxic - Beech Oxic	34.703	21.133	53	1.642	0.966	1.648	0.221	16	7.466	**<0.001**
	Maple Anoxic - Maple Oxic	-21.219	27.689	58	-0.766	1	1.408	0.221	16	6.378	**<0.001**
15d	Alder Anoxic - Alder Oxic	46.303	21.133	53	2.191	0.736	0.477	0.186	15	2.567	0.166
	Alder Anoxic - Beech Anoxic	72.983	21.133	53	3.453	0.08	0.578	0.172	15	3.357	**0.041**
	Alder Anoxic - Beech Oxic	77.72	21.133	53	3.678	**0.046**	-1.175	0.186	15	-6.316	**<0.001**
	Alder Anoxic - Maple Anoxic	53.91	22.36	55	2.411	0.587	0.026	0.186	15	0.140	1.000
	Alder Anoxic - Maple Oxic	32.673	21.133	53	1.546	0.98	0.220	0.172	15	1.279	0.791
	Alder Oxic - Beech Oxic	31.417	21.133	53	1.487	0.987	-1.652	0.199	15	-8.309	**<0.001**
	Alder Oxic - Maple Oxic	-13.63	21.133	53	-0.645	1	-0.257	0.186	15	-1.382	0.736
	Beech Anoxic - Alder Oxic	-26.68	21.133	53	-1.262	0.998	-0.101	0.186	15	-0.541	0.993
	Beech Anoxic - Beech Oxic	4.737	21.133	53	0.224	1	-1.753	0.186	15	-9.424	**<0.001**
	Beech Anoxic - Maple Anoxic	-19.073	22.36	55	-0.853	1	-0.552	0.186	15	-2.968	0.083
	Beech Anoxic - Maple Oxic	-40.31	21.133	53	-1.907	0.888	-0.358	0.172	15	-2.078	0.348
	Beech Oxic - Maple Oxic	-45.047	21.133	53	-2.132	0.774	1.395	0.186	15	7.500	**<0.001**
	Maple Anoxic - Alder Oxic	-7.607	22.36	55	-0.34	1	0.451	0.199	15	2.270	0.264
	Maple Anoxic - Beech Oxic	23.81	22.36	55	1.065	1	-1.201	0.199	15	-6.039	**<0.001**
	Maple Anoxic - Maple Oxic	-21.237	22.36	55	-0.95	1	0.194	0.186	15	1.044	0.895
25d	Alder Anoxic - Alder Oxic	42.343	27.689	58	1.529	0.983	2.793	1.322	17	2.113	0.326
	Alder Anoxic - Beech Anoxic	64.422	27.77	58	2.32	0.651	0.874	1.224	17	0.714	0.977
	Alder Anoxic - Beech Oxic	37.782	27.689	58	1.365	0.995	1.002	1.224	17	0.818	0.960
	Alder Anoxic - Maple Anoxic	7.25	21.133	53	0.343	1	-4.265	1.224	17	-3.485	0.028
	Alder Anoxic - Maple Oxic	29	21.133	53	1.372	0.994	0.515	1.224	17	0.421	0.998
	Alder Oxic - Beech Oxic	-4.561	32.966	58	-0.138	1	-1.791	1.322	17	-1.355	0.751
	Alder Oxic - Maple Oxic	-13.343	27.689	58	-0.482	1	-2.277	1.322	17	-1.723	0.536
	Beech Anoxic - Alder Oxic	-22.079	33.034	58	-0.668	1	1.918	1.322	17	1.451	0.697
	Beech Anoxic - Beech Oxic	-26.64	33.034	58	-0.806	1	0.127	1.224	17	0.104	1.000
	Beech Anoxic - Maple Anoxic	-57.172	27.77	58	-2.059	0.816	-5.139	1.224	17	-4.200	**0.007**

Beech Anoxic - Maple Oxic	-35.422	27.77	58	-1.276	0.997	-0.359	1.224	17	-0.293	1.000
Beech Oxic - Maple Oxic	-8.782	27.689	58	-0.317	1	-0.486	1.224	17	-0.397	0.998
Maple Anoxic - Alder Oxic	35.093	27.689	58	1.267	0.998	7.057	1.322	17	5.339	**0.001**
Maple Anoxic - Beech Oxic	30.532	27.689	58	1.103	1	5.266	1.224	17	4.304	**0.005**
Maple Anoxic - Maple Oxic	21.75	21.133	53	1.029	1	4.780	1.224	17	3.906	**0.012**

Statistically significant p-values (< 0.05) values are shown in bold

Table S8. Result of the linear model to observe the effects of FPOM type, leaf species, and conditioning on microbial growth efficiency (MGE). The model structure is MGE ~ FPOM type*leaf species*conditioning. Abbreviations; SE: Standard error.

**Factors**	***Estimate***	***SE***	***t-value***	***p-value***
FPOM typeLeaves	-12.048	5.043	-2.389	**0.022**
ConditioningOxic	-7.305	5.638	-1.296	0.204
Leaf speciesBeech	12.670	5.638	2.247	**0.031**
Leaf speciesMaple	-0.175	5.638	-0.031	0.975
FPOM typeLeaves:ConditioningOxic	6.710	7.132	0.941	0.353
FPOM typeLeaves:Leaf speciesBeech	-12.901	7.132	-1.809	0.079
FPOM typeLeaves:Leaf speciesMaple	0.910	7.297	0.125	0.902
ConditioningOxic:Leaf speciesBeech	-0.485	7.973	-0.061	0.952
ConditioningOxic:Leaf speciesMaple	1.472	7.973	0.185	0.855
FPOM typeLeaves:ConditioningOxic:Leaf speciesBeech	2.833	10.086	0.281	0.780
FPOM typeLeaves:ConditioningOxic:Leaf speciesMaple	-2.039	10.203	-0.200	0.843

Statistically significant p-values (< 0.05) values are shown in bold

Table S9. Microbial growth efficiency (MGE) measured in leaves at start (n = 29) and in faecal pellets collected at different time points (n = 18) during 25 days of feeding experiment. Mean and standard deviation (SD) are shown.

**FPOM type**	**Leaf species**	**Conditioning**	**Mean**	**SD**
Faecal pellets	Alder	Anoxic	12.71	11.64
	Alder	Oxic	5.41	2.21
	Beech	Anoxic	25.38	21.49
	Beech	Oxic	17.59	14.67
	Maple	Anoxic	12.54	3.79
	Maple	Oxic	6.70	1.33
Leaves	Alder	Anoxic	0.66	0.19
	Alder	Oxic	0.07	0.05
	Beech	Anoxic	0.43	0.07
	Beech	Oxic	2.19	0.51
	Maple	Anoxic	1.40	0.29
	Maple	Oxic	0.24	0.07

Table S10. Proportions of different fatty acids measured in leaves at start (n = 12) and in faecal pellets collected (n = 17) during 25 days of feeding experiment. Mean and standard deviation (SD) are shown. Abbreviations; ALA: α-linolenic acid, BFA: sum of all bacterial fatty acids, n-3 PUFA: omega-3 polyunsaturated fatty acids, n-3 SC-PUFA: omega-3 short chain polyunsaturated fatty acids.

FPOM type	FA_type	Conditioning	Mean	SD
Leaves	ALA	Anoxic	9.23	3.92
		Oxic	9.54	4.72
	BFA	Anoxic	5.94	4.18
		Oxic	1.99	0.47
	n-3 PUFA	Anoxic	10.54	5.05
		Oxic	10.00	4.69
	n-3 SC_PUFA	Anoxic	9.30	3.99
		Oxic	9.67	4.79
Faecal pellets	ALA	Anoxic	1.73	1.91
		Oxic	1.11	0.85
	BFA	Anoxic	11.22	6.34
		Oxic	12.37	8.01
	n3PUFA	Anoxic	1.99	1.78
		Oxic	1.58	1.23
	n-3 SC_PUFA	Anoxic	1.73	1.91
		Oxic	1.14	0.79
